# The graduate entry generation: a qualitative study exploring the factors influencing the career expectations and aspirations of a graduating cohort of graduate entry dental students in one London institution

**DOI:** 10.1186/1472-6831-11-25

**Published:** 2011-09-24

**Authors:** Paul Newton, Lyndon Cabot, Nairn HF Wilson, Jennifer E Gallagher

**Affiliations:** 1 King's College London Dental Institute at Guy's, King's College and St Thomas's Hospitals Oral Health Services Research & Dental Public Health Caldecot Road Denmark Hill London SE5 9RW UK; 2 University of Greenwich Centre for Nursing and Healthcare Research Southwood Site Avery Hill Campus Eltham London SE9 2UG UK; 3 King's College London Dental Institute at Guy's, King's College and St Thomas's Hospitals Restorative Dentistry Floor 25, Tower Wing Guy's Hospital London SE1 9RT UK; 4 King's College London Dental Institute at Guy's, King's College and St Thomas's Hospitals Office of the Dean and Head of Dental Institute Floor 18, Tower Wing Guy's Hospital London SE1 9RT UK

**Keywords:** dental, graduate, graduate-entry, career, motivation, workforce

## Abstract

**Background:**

Dentistry in the UK has a number of new graduate-entry programmes. The aim of the study was to explore the motivation, career expectations and experiences of final year students who chose to pursue a dental career through the graduate entry programme route in one institution; and to explore if, and how, their intended career expectations and aspirations were informed by this choice.

**Method:**

In-depth interviews of 14 graduate entry students in their final year of study. Data were transcribed verbatim and analysed using framework analysis.

**Results:**

There were three categories of factors influencing students' choice to study dentistry through graduate entry: 'push', 'pull' and 'mediating'. Mediating factors related to students' personal concerns and circumstances, whereas push and pull factors related to features of their previous and future careers and wider social factors. Routes to Graduate Entry study comprised: 'early career changers', 'established career changers' and those pursuing 'routes to specialisation'. These routes also influenced the students' practice of dentistry, as students integrated skills in their dental studies, and encountered new challenges.

Factors which students believed would influence their future careers included: vocational training; opportunities for specialisation or developing special interests and policy-related issues, together with wider professional and social concerns.

The graduate entry programme was considered 'hard work' but a quick route to a professional career which had much to offer. Students' felt more could have been made of their pre-dental studies and/or experience during the programme. Factors perceived as influencing students' future contribution to dentistry included personal and social influences. Overall there was strong support for the values of the NHS and 'giving back' to the system in their future career.

**Conclusion:**

Graduate entry students appear to be motivated to enter dentistry by a range of factors which suit their preferences and circumstances. They generally embrace the programme enthusiastically and seek to serve within healthcare, largely in the public sector. These students, who carry wider responsibilities, bring knowledge, skills and experience to dentistry which could be harnessed further during the programme. The findings suggest that graduate entry students, facilitated by varied career options, will contribute to an engaged workforce.

## Background

### Context of Study

In the United Kingdom undergraduate dental students have traditionally emerged from typically five, or possibly more, years of education and training in dental schools to become part of the professional healthcare workforce. In recent years the environment into which these students emerge has been of one of rapid change, particularly in England - characterised by expansion of students numbers [1]; a shift to the local commissioning of dental services [2,3]; increasing privatisation [4]; formalisation of specialisation [5], and special interests [6,7]; as well as an emphasis on the appropriate use of the dental workforce skills mix in oral health provision [8-11]. Whereas in the early part of this decade, dentists were considered to be in short supply, subsequent actions by the Secretary of State to recruit internationally and expand dental student numbers [12], together with net workforce gain through global migration [13], and the reduced flexibility for the workforce imposed by the new dental contract has meant that capacity has increased and there may be fewer openings for newly qualified dental health professionals. Furthermore, few professional groups will be immune from current global economic conditions and this is likely to have ramifications for young dentists entering the profession and their future career plans.

### Rationale of Study

Concurrent to the above, the increase in the ascribed numbers of training places for dentists has led to the development of a number of graduate entry programmes. Graduate entry programmes have become commonplace in the UK and beyond particularly in medicine [14,15], dentistry [16], and nursing [17]. Graduate entry programmes encourage applicants who hold a higher classification of degree, and match a set of criteria determined by the institution, to study to become a healthcare professional. In the United Kingdom's dental education system graduate entry programmes are designed to meet the needs of the dental workforce in two ways. First, these programmes can be seen as time and resource efficient since graduate entry programmes take fewer years to complete. King's College London Dental Institute [KCLDI] is just one of a number of schools across the UK which offer offers a four year course for graduates holding a degree (the Dentistry Graduate/Professional Entry Programme) [16], the first tranche of which graduated in the June 2009. Second, it is anticipated that graduate entry programmes are also designed to contribute to improving access to education and training through broadening access to dental training, and, in turn, diversify the supply of students and professionals by background (mostly in terms of age and socio-economic background). Studies have noted that, traditionally, medical and dental students have been selected on the criterion of academic success; hence, the 'traditional' route students have taken has lead to a tendency for dental graduates to have similar socio-economic characteristics, and for their routes to study to be relatively homogenous [18]. There is, however, some debate over to what extent diversification of students may occur as a result of graduate entry programmes in nursing [19,20], and medicine [15,21-24], and the benefits of such.

Research into career motivation, expectation and aspiration for studying dentistry covers a broad range of factors [25-31], and are important for workforce planners and policy makers to understand [32]; these studies are also becoming increasingly important in demonstrating that what dental students (and practitioners) want and expect from working life and career development can inform, and have ramifications for, workforce retention and thus planning [9].

There is limited evidence that graduate entrants to medicine have widened the academic and socio-demographic diversity in the medical school student population in a variety of countries - including the UK [33]. If the same is true in dentistry, there may be ramifications not only for the support required during the educational programme, but also - given that this cohort's route to studying dentistry is different - they may bring new and varied motivation, as well as having different factors that influence their future career aspirations and expectations [20,21]. The premise of this study is that the varied route to dentistry, combined with a diversity of influences and motivations of graduate entry students from diverse backgrounds, will have implications for dental education and workforce planning. Therefore, prior to the graduation of the second cohort of students who gained entry through the KCLDI graduate entry programme, this study sought to explore graduate entrant students' motives for, and experiences of studying through a graduate entry programme. Concomitant with this, the study also sought to explore how taking a different route to dentistry informed graduates' interests and concerns as they embarked on their dental careers.

The aim of the study was to explore the experiences of final year students of KCLDI who chose to pursue a dental career through the graduate entry programme route; and to explore whether (and if so, how) their intended career expectations and aspirations were informed by their decision to enter dentistry through the graduate entry route.

The objectives adopted in supporting this aim were to:

1. Explore students' motivations for choosing dentistry as a career, and the route they took to realise their motivations.

2. Explore the career expectations that they had for their professional working lives as dentists in both the short-term and long-term.

3. Identify the factors, which - students perceived would - influence their short-and long-term contribution to the dental profession; and how their personal concerns, commitments and career expectations, and aspirations, impacted on and shape their choice, and capacity, to contribute.

4. Identify if there was a level of certainty about future career plans and whether and when this emerged during the professional course; and what factors within and outside the field on dentistry have impacted on their plans; as well as how these concerns were appraised, prioritised and managed by the student participants.

5. And ultimately - through elucidating their experiences and reporting the issues facing graduate entry students about to enter the workforce - inform the professional leadership, policy makers and providers of dental care on issues of future workforce decision-making pertaining to this group.

## Methods

### The qualitative approach

As outlined above, this study sought to capture the views of a small, newly emerging group whose views are nascent and remained relatively unexplored. Although the graduate entry students' views may concur with existing understandings and findings of respondents in previous studies [28-31,34], how they arrived at their choices may vary (due to the route they have taken and their differing backgrounds) - this would have, for example, important implications for motivation. Green and Britten [35], advocate the use of qualitative research where very little is known about a subject area, and where researchers wish to inductively generate data and explore in-depth understandings orientated around the participants' understandings. As it was the unexplored, nascent data this group may offer and that the research methods wished to capture and explore - "The 'what', 'why' and 'how' questions about [the] phenomenon" [36] - the qualitative approach emerged as most suitable. Added to this, it is commonplace for qualitative approaches to employ purposive sampling to locate small homogenous group, as this allows for the range of experiences regarding choices, preferences and motivations of participants to emerge and be explored [37]. In short, given the small sample (n = 22), and the need for exploratory in-depth, inductive data - a qualitative approach was the most suitable method to address the aims of this study. It has previously been successfully employed within the KCLDI to explore dental students' career motivation and career progression [30,34], and in wider healthcare settings [38,39].

### Study design

**Figure 1:**
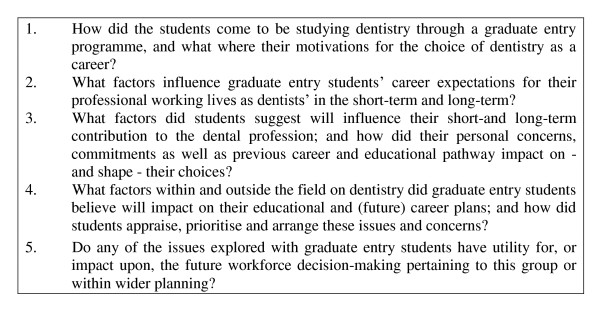
**Topic Guide: key areas explored**.

Following ethical clearance by Kings College Research Ethics Committee (Ref: BDM/09/10-22), 14 of the total 22 graduate entrant students in their final year of study were recruited through email circular, and interviewed using structured one-to-one interviews. Participants were offered a small honorarium in acknowledgement of the time they had given. The data were collected over the period of October 2009 to January 2010 and the recorded interviews were transcribed verbatim. The areas covered in the topic guide for interviews related to five key research questions derived from the aforementioned research objectives; and as they contributed the overall aim of the research (Figure 1). Questions 1-4 related directly to participant reporting. The final question was asked indirectly through questions pertaining largely to what wider factors participants felt would influence their careers, thus the relationship and fit with workforce planning was analysed and collated by the researchers - post-facto. This was enabled by employing framework analysis, a method of data analysis widely used in health-related and policy studies [37].

Framework analysis allows for systematic and visible stages to the data analysis process [37]: familiarization; identification of a provisional thematic framework; indexing; charting; and mapping and interpretation. Framework analysis follows a standard 5 stage process. In the initial familiarisation stage transcripts were read and re-read, and key emerging themes and ideas were listed framed by areas of the topic guide. A provisional thematic framework identifying key issues, concepts and themes was developed, so that the data could be examined and referenced to themes through coding. In the data indexing process, the researcher applied the index of the thematic framework to the transcriptions. During the charting process the data were rearranged through constant comparison, and the thematic framework was expanded in light of the application of the data. During the mapping and interpretation stage, the data were the synthesised information generated from indexing and charting to define concepts; map the range and nature of phenomena as well as find patterns and associations. In this stage the aim is to provide explanations for the findings, and generate theory [40].

## Results

The results are presented in five sections below, broadly in line with the objectives, starting with students' motivation for studying dentistry and route into the programme. An overview of the results is presented in Table 1.

**Table 1:** Specific and general factors perceived to influence the maximisation or minimisation of contribution to dentistry in general, private practice and NHS-related dentistry

**Factors minimising contribution**	
**General factors that were seen to have a minimising effect on contribution to** ***dentistry***:	• Perceived ability to mitigate current/potential family and work life balance that that are available • Job security and work-related benefits that that are available • Perceived potential to specialise and for training that are available • European Time Working Directive
**Specific factors that where were seen to have a minimising effect on contribution to** ***private practice***:	• Private system seen as inequitable • Lack of challenge and opportunity (patients are, on the whole, well) • Whether there will be time for private practice when established in an NHS career
**Specific factors that where were seen to have a minimising effect on contribution to the** ***National Health Service***:	• Units of Dental Activity as disincentive for NHS work. • Perception that NHS limits ability to practice. • Hospital-base seen as less autonomy and poorer quality of working life (although willing to work in NHS general practice).
**Factors maximising contribution**	
**General factors that were seen to have a maximising effect on contribution to** ***dentistry***:	• Perceived ability to mitigate current/potential family and work life balance that are available • Job security and work-related benefits that are available • Perceived potential to specialise and for training available • Clear career trajectories (Particularly in hospital setting)
**Specific factors that where were seen to have a maximising effect on contribution to** ***private practice***:	• System seen to offer a more profitable financial package and greater potential earnings • More time and resource to meet patient need • More autonomy of practice and more instances to apply and develop greater technical skill. • Ability to be an independent practitioner.
**Specific factors that where were seen to have a maximising effect on contribution to the** ***National Health Service***:	• Greater amount and clarity of career trajectories as well as opportunities to train and specialise (Particularly those specialising or seeking a hospital-base) • Job security and work-related benefits in NHS • Support of principle of the NHS & 'Making a difference' • Ability to be an independent practitioner (in general practice) and determine own workload

### Motivation for studying dentistry: 'push', 'pull' and 'mediating' factors

In the early part of the one-to-one interviews students were asked a series of questions with regard to their motives for choosing dentistry as a career. In analysis, it emerged that students reported experiencing a variety of 'push factors' relating to their previous careers, and a variety of 'pull factors' relating to dentistry as a career - both of which influenced their motivation towards a career in dentistry. 'Push factors' related to both the day-to-day and institutional circumstances of their previous careers including: dissatisfaction with the pay and working conditions in their previous careers; a lack of personal fulfilment as well as a lack of career development in their previous careers/degree.

'I specialised in biochemistry and essentially was going out into labs and pressing buttons and I thought I haven't got to do [Laughs] a three year science degree to do that, and so that's when I pretty much ... when I decided that I was going to apply to do dentistry' R2: 46-50

'I was working [...as a pharmaceutical manager...], and in order to progress with work I needed to do a PhD or a Masters. So I kind of looked into the courses PhD's and Masters, they were about four years so it just didn't make any sense - and I kind of just pieced together dentistry really' R7:37-41

Concurrent with this, were 'pull factors' in dentistry such as a wide array of career development options; having the opportunity to be an independent practitioner; as well as dentistry being a 'patient oriented' and practical 'hands on' career.

'I enjoy patient interaction, I like meeting new people - when I was doing my first degree which was just in the lab I really didn't enjoy it, it was just the same thing day in, day out. So I definitely wanted to meet different people all the time and I wanted it to be ... sort of like a healthcare professional. I prefer dentistry because it's more on a regular basis, you'll see the same patient and its nine to five.' R12:76-83

'Dentistry just seemed sort of the more likely option for me for the first reason that it was a lot more practical, I do enjoy sort of that aspect of it' R2:97-99

'I suppose I was looking to be sort of more of an independent practitioner [in healthcare]. ' R1:67-82

As we can see, however, the decision to act on these 'push' and 'pull' factors was also mediated by the students' personal concerns and circumstances. Hence, students discussed assessing these 'pushes' and 'pulls' through personal factors such as improving the quality of their life; in terms of balancing family and career; as following a long-standing interest in oral-health; as well as attaining job satisfaction and financial security (demonstrated passim below). Students gave well reasoned and thoughtful accounts of how aspects of a career in dentistry suited their preferences and circumstances. In many cases the, frequently negative, aspects of 'push factors' that had influenced their decision to change career, had also influenced the array of criteria that they sought or expected in their new career. This process is demonstrated succinctly by this respondent, who continues:

'So dentistry pretty much ticked all the boxes because it's - pretty much - Monday to Friday... what I really loved about my job in [healthcare] [...] was the fact that it was about my patients, and the clinical work. And dentistry very much - well, its clinical work and it's with patients. What was appealing as well was that at some point, maybe, I might have a bit of autonomy if I was looking at general - working in general practice. I was tired of shift work and I think my family were too [...] And then probably about - sort of number five on the list - would have been some security for the future.' R1:84-96

Here we can see that the option of practising as an independent practitioner in a fixed working pattern is appealing as it addresses the respondents' concern with the unsociable hours and the stresses that her previous career had placed on her family life and quality of life.

Although, although these 'push', 'pull' and 'mediating factors' resonate throughout all the respondents' interviews, for a few participants the overriding motive for studying dentistry was to specialise in maxillofacial surgery. Hence, although these factors came into play, the dental degree was a *means *to a longer term *end *of specialisation. Hence, their primary 'pull factors' were couched in terms of choosing a specialism. Overall, these respondents argued that maxillofacial surgery as a specialism constituted a 'cutting edge' aspect of surgery and represented a unique body of knowledge and expertise - and that this drove their interest.

'I wanted to train in Maxillofacial surgery... European Law mandates you need a medical and a dental undergraduate degree [...] I needed to return to dentist school from a career as a doctor [...] It's the most exciting branch of surgery I've experienced in my training. It's a combination of attention to detail, familiarity with the techniques that are to do with the aesthetic finish of surgical work not just the functional features And - erm - it's a very varied speciality as well. It uses lots of different surgical techniques and I like the variety angle.' R3:42-44/46-50

Those who chose their previous degree/career after having not secured a place at a dental school reported the graduate entry programme itself was a sufficient 'pull factor' to consider dentistry again, with the added push of feeling a lack of fulfilment in their previous career. The remainder of students, however, actively selected dentistry from an array of options (medicine being the most common alternative) mediating between how the characteristics of a dental career suited their preferences and circumstances. In short, the decision process for the graduate entry route to dentistry was three fold: those who followed a long term interest awaiting the opportunity to study; those to actively selected dentistry as a career change from an array of options; and those for whom it was it was a necessity of specialisation in their future career.

### Graduates' routes to dental education: early career change, established career change and pursuing career specialisation

The students interviewed were also asked a series of questions relating to how they came to be studying dentistry through a graduate entry programme. Clear routes to dental training were evident in the Graduate Entry Programme [GEP] students interviewed. The routes were defined by their motive for, and route taken to studying dentistry and in terms of time elapsed between their previous and present degree. First were those who were in what would be considered in the early phase of another career and had decided to train as a dentist. This occurred where students had trained in another area (mainly Bio-Medical Science or Pharmacology), before moving on to dentistry. The gap between their degrees was short - with some students saw their early degree as a stop-gap - maintaining the hope of studying dentistry at a later date. This was done for a range of practical reasons to put the students in a position to apply, such as when they had not achieved the necessary A-Level grades.

'To be totally honest, it [biomedicine] was a means to an end for me. I enjoyed, in the final year I did an oral biology which was a lot of overlap with me for second year, so that came in and that helped me then. I also did a project on dental implants and their development, but other than that the others were very small modules, that made up over two thirds of my year, and that did help me in this, but everything before that, harsh as it is, how shall I say, the degree itself was always a means to an end for me' R10:307-314

The majority of this group stated they were following a long-term interest in oral-health, and a desire to work with patients; however, some of this grouping had worked briefly in these nascent careers, and reported these periods of work as being unfulfilling or lacking career options.

'I mean there's no patient involvement in a laboratory which is something I desired quite a lot, and I realised it wasn't there.' R8:73-74

The second route comprised of those who broke from an established career to train as a dentist. All came from a healthcare background (e.g. nursing and pharmacology) their motives often related to the attractive career opportunities available in dentistry. In seeking to escape a career with poor pay, lack of career development and so forth, the opportunity and flexibility of being an independent practitioner with a variety of career options fitted their personal requirements of improving quality of life, managing work-life balance, and so forth; however, contrary to the 'early career change group', many still reported enjoying, and even missing their previous work.

The final group, were those who undertook a dental degree, following medical training, and solely with the purpose of specialising in maxillofacial surgery (a surgical specialty). Here the students' motives, although subject to similar 'push' and 'pull' factors, extended outside the 'push factors associated with their previous career' and beyond the 'pull factors of dentistry'. Ironically, their approach to studying dentistry resonated with the 'early career-changers' attitudes towards their first degree.

'I do it because I have to, to do the career that I want to do. There's no sort of great passion for dentistry unfortunately.' R14:71-72

Evidently, those specialising and coming from an established career were older - frequently with family commitments, whereas those with shorter periods between degrees tended to only be in their later twenties (closer in age to the majority of their dental student peer group). As is evident in the relevant quotes above, the older group tended to focus of improving quality of personal and working/family life and career satisfaction as personal motives for studying dentistry. For younger students the acquisition of new knowledge and skills as well, as more 'instrumental' aims of financial security and establishing a long term career trajectory were placed in the forefront of their motivation for studying dentistry.

### Influence of routes on practice of dentistry: Bringing skills, integrating skills and challenges encountered in changing careers

The students interviewed were also asked whether their background and previous careers had influenced their study and practice of dentistry in any way. Students reported the route they had taken to study positively informed their approaches to dentistry. The graduate entry programme students interviewed reported bringing practical skills such as study techniques from their prior degree/s, case management and patient skills to their dental studies and practice. They also reported bringing skills in research and working as part of a team (the latter being especially relevant where their previous degree was healthcare related). The students also felt that the technical knowledge acquired in their previous discipline, and their knowledge of the overall workings and structure of the health service (albeit in a discreet area such as pharmacology, nursing/medicine or bio-medical testing) gave them an added, and/or adaptable, set of skills in the practice of dentistry. Many also felt that these skills were not only pertinent in a study environment, but would become more pertinent in a 'real' practising environment.

'I think it helped me a lot actually because I think I learned a lot more in my first degree, learned a lot more about my work ethics, how to apply myself to revision, how to apply myself to my studies, of what really it entails at university rather than just at A-levels and GCSE. So I had a much broader grasp of what university really wanted from you and demanded from you to, in order to get through.' R10:55-62

*'My nursing experience has helped me as a student in dentistry. Erm..., I really think some aspects do, I certainly think being at ease with patients, I don't worry about patients, or difficult patients, or ... difficult situations - things like that.' R1:109-112*.

In terms of integrating skills, those specialising and leaving an established career reported a process of integrating their previous skills into their practice. Those from healthcare backgrounds (as shown above) reported being comfortable with patients and with working within a team which they considered integral to good practice; however, many of those with a bio-medical degree felt there was no clear means to integrate their knowledge of their old and new disciplines.

'I mean, yes, there are elements of my previous degree that I do definitely apply to dentistry and especially - I mean the immediate one that comes to mind is things like oral medicine and having a better knowledge than sort of your average undergraduate about sort of taking bloods and what they're for and that sort of thing. But outside of that I don't think it's actually had a massive impact on the actual sort of practising of dentistry in that sense.' R2:58-64

Whereas, those specialising in maxillofacial surgery described an ongoing process of adjustment and of weaving both medical and dental disciplines together to fashion their skills and knowledge to the needs of their future discipline.

Respondent: *'Doctors have a habit of avoiding the black hole in the lower half of the face [...] So there's an extra chunk of knowledge that I'm adding to my medical theoretical knowledge. I am only just beginning to see the value of integrating dental theory and dental practice to my medical theory and medical practice. They are different. I'll give you an example. Doctors talk about history first - then the examination. Dentists put a lot of emphasis on the examination - and relatively little to somebody's history, and in terms of going about your treatment and understanding what's happening ... understanding what's happening in an efficient way - I think there's even less in common in both approaches. And it's not straight forward to integrate. I do think maxillofacial surgeons offer something unique for that reason.'*

Interviewer: *'So does maxillofacial draw them both together?'*

Respondent: *'We say, when done well, it does.' *R3:74-97

Respondents, however, specialising and having previous established careers reported challenges in changing careers particularly with regard to overcoming their previous professional socialisation, and often involving a return to a 'junior' status on entering the dental programme.

'I'm a nurse, and I come with a culture of a nurse as well, whereas a doctor will be ... you know, I don't know if they're more confident about themselves because they are doctors, and as a nurse I'm not used to being that confident. Well, confident in the nursing things but you always feel that you're working under somebody, or you're below somebody, so I don't know if [...] I've put that hat on and that monkey on my shoulder and I've held myself back a little bit by being a bit ... you know, still having my nurses' hat on' R1:438-448

'I mean the dental faculty don't understand us as doctors as such. I mean that, that it is not only at a professional level - you know like nurses would treat us differently [...] I think one maybe like er, they don't understand what we're doing and sometimes they don't know what our capability is. And then probably, you know, like, there is a professional conflict. They think that we're going to do it wrong so like you have to correct us and they don't understand what we are capable of actually' R6:155-166

With those specialising and moving from an established career this frequently entailed managing an academic workload, whilst often working part-time and having family commitments. Many felt that the needs of mature students were overlooked. Many respondents stated that being in a youthful, bright peer group who had fewer responsibilities entailed accepting that it was impossible to excel, or 'shine'. Also, many felt that the circumstances of mature students were overlooked.

'[The Dental School] ... is looking after a bunch of fairly open minded 19, 20 year olds and they're only commitment is dentistry. And it's very frustrating that because[...]the dental school doesn't really acknowledge the different needs of mature graduates who have outside responsibilities, be it to their original calling, or their family or paying the mortgage.' R3:299-304

These respondents also felt that they had excelled in the social and behavioural components of the course, and that their skills would be more apparent in application and practice.

In short, many students brought a range of skills which they actively sought to integrate into a new career within or associated with dentistry. Those with a bio-medical background tended to 'park' the skills associated with their previous career. Older students often found it difficult to adjust to the demands and culture of dental study, and frequently felt that their unique set of concerns and circumstances differed from those who had taken a more traditional route to dentistry. Mature students felt that their route to dentistry had positively informed their day-to-day practice and study of dentistry, however this also brought a different set of circumstances and concerns (as demonstrated above), which were in tension with the intensive dental programme and were not addressed, at the faculty level.

### Factors which students believed would influence their future careers: Vocational training as an establishing period; specialisation or developing special interests; policy issues; personal and social concerns

Respondents were asked a set of questions relating to their vision for their professional lives - relating to the nature and mode of work they were considering; and what factors they used to appraise their choices. Thematically, four key areas emerged: Vocational training as an establishing period; specialisation as both a career path and as an influence in wider workforce trends that would inform their careers; the influence of planning and policy issues in dentistry and wider health; as well as personal and social influences which would inform their future career direction.

For those embarking on a vocational training (VT) year, this year was seen as a period in which to develop a practical 'sounding board' with which to address these issues. In this manner, the VT year was held as a period of time to allow opportunities and interests to arise by their assessing skills, capacities and preferences; whilst also being engaged in assessing 'the practice sides of things':

'I'll have (at the end of VT) - you know - got some experience and some confidence under my belt.' R1:285-293

'I think it's just experiencing the practising side of things. Of course you get your opinions based on what other people have told you of what it's going to be like during your VT year, or when you're practising as an NHS dentist. That will have a big bearing definitely on me in the sense that if I feel like I'm under a lot of pressure, or you know, if I'm not enjoying the particular practice I'm in and that sort of thing. I think that, generally, will play into things as well. ' R2:213-226

Many students were also considering - or intending to complete - the optional extra foundation training year to secure the option of specialisation. In analysing the interviews it became apparent that this group of students followed a similar process in deciding to specialise.

First, the students reported that in deciding to specialise they had developed, or felt they wanted need to develop, a special interest in an aspect of dentistry - or related practices - that they found interesting and rewarding (i.e. in an auto-didactic way).

'I think I just enjoy the anatomy - [...] ... the functions that you're dealing with, people's appearance, their speech, their swallowing, they're all pretty vital things for interacting with other people and there isn't really any ...[...] although plastics has a bit of crossover, it doesn't really do the kind of ... the hard tissue stuff [...]. It's nice to be able to interact with some of the other specialties orthodontics and so on. Yes, I guess I just ... I just erm enjoy the surgery a lot more than any, any other type that I did.' R2:149-157

Even where this was established, however, the students were still faced with the decision of choosing how much of their career to invest in each specific area, and when to develop this special interest.

'Yeah I mean in Special Care [new specialist area of dentistry] I just feel it's a really rewarding, But I think with me, I kind of know the kind of person I am, and I think working in a practice, just doing dentistry would be great, I would be happy with that ... but it would be nice to have an added interest.' R7:150 -154

'For me, I will probably like to specialise myself, not immediately, I've done enough of studying for the time being, but I would say within between five and ten years I'd like to go, come back and specialise, [...] at the moment I would say endodontics - I really enjoy, and then later in life I might come back again and specialise further in something [...] like orthodontics I find very interesting. But I think I'd like to do that later on when I've had my enjoyment of the general dentistry, 'cos I know I want to do. I thoroughly enjoy every aspect of the dentistry I do, but erm, even if I did specialise I don't think I'd ever stop being a general dentist.' R10:118-129

Secondly, all who were considering specialisation were aware that some practical and prolonged engagement to the ends they sought was necessary, and they may have competing concerns once they were in practice. More practically the students' interests in specialising were considered in light of the roles available for them to pursue their aims.

'I mean within the bio-medical field you essentially ... I mean you did have options to the wider career paths, you could always go into law if that was related to healthcare and that sort of thing, but the essence of bio-medical science meant that there was four specialities you could go into [...] Within dentistry not only do you have the difference between sort of secondary and primary care, but then also specialising as well, you can just go into so many sort of specifics with dentistry in that sense [...]. I do think is important to me is that, I guess why I don't wanna specialise so quickly is that I don't want to sort of cut myself short in that sense, but there is always the worry that things will change and you don't get the opportunity to specialise.' R2:243-249

Finally, students acknowledged that the process would involve integration of various bodies of knowledge within the intended specialism. In this sense, specialisation was seen as a personification of an extant role with a unique set of skills and interests that they had developed. This meant that they would have a range of interesting cases and have the opportunity to explore aspects of specialisation that interested them.

'I mean in Special Care [new dental specialty for people with special needs] I just feel it's a really rewarding, I mean we're doing it today funnily enough, and you just see a large diversity of patients, you're still doing a lot of the main kind of treatments but you're seeing a different range of people, and there's different challenges associated with each person, and [...] for oral surgery as well, there's erm, you've not just got the extractions, you've got the oral medicine part and I guess it overlaps a little bit with the cancer care as well. So there,[...], it's not monotonous, you've got a range of things that you could be doing. [...] But I think with me, I kind of know the kind of person I am, and I think working in a practice, just doing dentistry would be great, but it would be nice to have an added interest.' R7:142-154

Although the students were practical in using their VT year as a sounding board for their skills and expectations, students were also aware that practical personal; social and policy issues would be impacting upon and influencing their career choices, and their ability to realise their expectations and aspirations (as will be discussed below). Hence, although having a tangible role which reflected their specialisation was an important visioning factor, specialisation *as a wider process *- within the milieu of the dental workforce - was also considered to be a significant factor for students when considering their future careers. In particular, how teams and skills-mixes in the workforce as well as trends in evidence-based medicine would influence the roles and responsibilities available (or conferred) to them.

'I think also one thing that would inform the ... err my professional outlook is that we will work in teams more out of necessity, the training opportunities don't allow me to feel so confident at such an early stage to take responsible decisions on my own. And there'll be a safety in numbers culture developing where nobody is prepared to say "I'll see what I can do and this is where we're going into the unknown" - not because there is evidence for this or that. It will always be a case of "Well we've discussed it all as a team and this is what a common way - we agree in a consensual way". And I think, you know that's 21^st ^century practice and that fits nicely into this heightened sense of [...] the legal err [Huh-uh] consequences of the decision making - and working from an evidence-base' R3:141-152

Added to this, policy trends such as reimbursement through the current 'currency' of units of dental activity (UDAs), and wider funding issues in the recession, as well as how the influence of evidence-based care, will influence how dental care at a macro level. Since April 2006, NHS dentists in England have been paid according to the volume of "Units of Dental Activity" they do in a year [3]. The actual cash value of a UDA is practice-specific under the influence of commissioners, and controversy remains about this system, to which changes are anticipated [41]; hence, students were concerned about reimbursement mechanisms that might impact on their contribution to the NHS.

*'I'm very supportive of the NHS, but erm it's, it's erm (pause) changing all the time isn't it and the...the government of the day has all sorts of ideas about how it should run and often based on targets which aren't always obtainable by the means that they think it can be achieved, so I mean it's a ... there's no guaranteed jobs now and erm so I guess that is going to be a big factor if there's cuts in certain departments then erm you know there might not be any opportunities' R14:171-179*.

'I think there needs to be more of a balance in the type of treatment that's delivered to NHS patients and also I think that the pricing system is quite a mess which is probably why there's not many NHS dentists. But that's what I think' R12:180-183

Factors which had informed the graduate entry students' motives and routes to study were also influential in their proposed career plans. Amongst their personal considerations were time for family life (in the present or future) and a work-life balance, and having a fulfilling role with career progression and financial security (as highlighted passim). The majority of graduate entry students also demonstrated that strong ethical principles guided and informed their career choices. The majority of respondents gave strong support to the principle of the National Health Service (NHS) as an equitable means of providing dental and wider health care and treatment. In all cases this was rooted in a strong commitment to the principle of dentists having an obligation and responsibility to provide oral healthcare to all. Many saw the NHS as a duty and responsibility for dentists, and thus an overriding principle within and of itself. The reasons proffered by respondents related to personal commitment and belief in the service. In terms of motivation, there was a strong reporting of working for the NHS as a social duty, and indispensible 'public good'. The following quote describes this succinctly:

'The more mature you are the more life experience you have, the more you may ... the more value you may place in the value that the NHS offers to most of the public - most of the time - particularly for emergency care and ...erm ... No, if you've ever had a sick child, if you've ever had a sick parent - you'll know that the NHS is there as a mother ship. It's not perfect but it does do most people - most of the time - a good service. And the public when asked will applaud that. As opposed to eighteen, nineteen, twenty year olds down the street, I can't blame them for thinking you know it's all down here, it's what I can get out of the system and having a slightly more egotistical view on things. But I think some of the guys here [GEPs] they've gone back to do a vocational degree because - and it is a vocational degree - because they see value in giving back to society and I would not be surprised that in more ... that a higher proportion of the graduate entrance students aspire to that, if not completely that sort of sentiment.' R3:273 -287

The majority of graduate entry programme students interviewed indicated that they wished to practice outside of London for reasons such as 'giving back' to the community; serving deprived populations; as well as their personal 'quality of life'. This is evidenced here by a respondent discussing her motives for wishing to enter general practice in the place she was raised:

'I'm hoping to apply for VT to go up back to [Place Name]. I'm lucky and I'm fortunate that I don't have sort of the commitments to keep me in London erm and because I just had such an enjoyable time in Yorkshire, I mean people are just friendly, there's all sorts of things that sort of were pluses for me that means that I can enjoy life a bit more. I'm just happy to go with where I feel comfortable really and so that's sort of the plan for now.' R2:199-206

### Factors influencing students' future contribution to dentistry: Personal and social influences and perceived mitigating factors; Hospital and general practice; National Health Service (NHS) and private practice

The students who participated were asked what factors would minimise and maximise their contribution to dentistry in general, and specifically to contributing to the NHS and private sectors. Many of the personal considerations that would influence their future contribution to dentistry or related professions - albeit in general, NHS or the private sector - or related to having a family life and/or work-life balance, and having a fulfilling and financially rewarding job. Many students (as seen above) added to this that the potential to specialise as well as the potential for career development would also influence their future contribution. Those planning to specialise in Maxillofacial surgery also cited the European Time Working Directive as a major influence of their ability to contribute to the workforce as it limits the hours of work possible, with trainee doctors limited to a 48-hour week [42]; however, many argued that in a hospital environment structuring activity around set hours was not always possible particularly in a surgical context.

Students were specifically asked what would minimise and maximise their contribution to the NHS and private practice. With regards to maximising contribution to NHS and private practice all respondents were intending to practise in the NHS in either a full or partial capacity.

'I think it's, it's more affordable for a lot of people, it gives patients more options to be able to come and see you and the whole point of doing dentistry is to be able to help people, erm, cos a lot of people can't afford private dentistry, so I still think, you know, the NHS is a good thing in that sense. Erm, so yes, but I also like the idea of being able to offer other treatments, like more complex treatments that wouldn't be available on the NHS - so that's why I think a bit of both' R5:85 - 90

Those specialising maxillofacial surgery noted that there was a clear pathway within NHS secondary and tertiary care. Others cited their commitment to the NHS (discussed above) as their primary motivation. Another strong theme running throughout the responses was the commitment to NHS dentistry. The key factors influencing this being a belief that dentists should 'take ownership of oral health'; a deep conviction to improving access to dentistry and arranging dentistry so dentists are able to meet the needs of the population as well as the practice of dentistry should encourage the provision of good quality of care.

There was, however, split between those who wished to work in a hospital or general practice setting. Many of those opting towards practising predominantly in a hospital setting emphasised the possibilities for training and specialisation available in this setting (particularly for those specialising). Those considering general practice tended to emphasise *not only *the control they would gain over their working life:

'Hospitals tend to take over your life...' R1:250

*But also *the flexibility and quality of life enhancing aspects that some respondents believed being an independent practitioner in general practice would incur (discussed in motives for choosing dentistry).

Although all respondents showed a strong commitment to the principle of working in the NHS at both the hospital and general practice level, some still wished to maintain a private practice portfolio (particularly in general practice). The factors minimising their contribution to the NHS related predominantly to what they perceived to be inconsistencies in the method of funding NHS dentistry at the time in England (Units of Dental Activity [UDAs] which were allocated to different types of courses of care).

'Scrap the UDA system and probably a greater emphasis on prevention as opposed to just treating - because I think the old system was quite lucrative for dentists and that makes it unfair. But if you obviously a balance that ends with dentists are being ...erm ...fair, and like offering good preventative advice then it's the best of both worlds really. They still have the answers and then when they do treat they get rewarded accordingly, but they're not just treating willy-nilly just to get good pay packets. [Laughs]' R4:254-261

Other considerations maximising a contribution to private practice were concerns that the NHS could potentially limit and circumscribe their ability to practice, and to use and develop their skills (some relating this to the current system of UDAs). Others extended this by arguing that it was possible to spend more time and resource on patients to greater effect in private practice. Other factors maximising the desire to work in private practice related to better earning potential as well as greater personal and practising autonomy.

'Personal development in the NHS is quite difficult because you will end up working in a practice for, it's quite easy to end up working in a practice for a lifetime, and sometimes that's what people want. You can be an excellent NHS dentist in a small village and have a beautiful practice on that basis, but you will stay there most of the time. Once you get into that routine I think it's quite difficult to get out at a later stage.' R8:208 -214

'The rewards they can get out of it (Dentistry), the freedom you get you out of practice I think would push my choice ... I'm assuming with NHS you might be a more restrictive the ability to have the freedom to be what you want - because it is working to a precise structure [...] Obviously helping people who can't like necessarily be able to afford the treatment elsewhere which is quite important because it's not ... obviously dentistry is not only about you know, giving like cosmetic to those people who can afford it.' R4:239 -247

Conversely, those who sought to practice solely in the NHS stated a belief that there was more support and opportunity for training and specialisation in the NHS - particularly in secondary and tertiary hospital-based dentistry. Both those intending to pursue a hospital or general practice NHS career also emphasised that there was an ethical principle to NHS working, with some even arguing that private practice did not challenge or enhance skills as the majority of patients able to afford private care have good dentition. Others supported their choice to work in the NHS by referring back to giving back' to the community and serving deprived populations.

In summary the general and specific factors maximising and minimising contribution to dentistry can be portrayed as follows:

### Being a Graduate Entry Programme student: Hard Work but a quick route to a varied career where Graduate Entry Students can bring new skills

Overall, with regards to their course the GEP students interviewed in their final year acknowledged that the content of the course was 'hard work'. Many also wished that there had been time and encouragement for more in-depth learning as they preferred to see the utility and application of learning as opposed to 'learning by rote'. All stated that they found the dental degree a challenging and difficult course to manage with other commitments requiring their time and resources (work and family life in particular). These, however, were minor points for students when compared to the positive aspects they reported. Students reported integrating quickly into their studies having joined in the other students 'second' year', and that the course was valued as a quick route into dentistry. Mature students also noted that the distractions of student social life were less appealing with age and with having experience of a career and/or family life. Many students interviewed acknowledged the positive aspects of diversifying the mix of dental students (discussed above), and that the skills they brought to the dental team were reflected in the wide range of career options available in dentistry, and would flourish in practice.

'My hope is that in two or three years time, if you look at us all, that I ... I ... I would put myself up with them [the bright students who pick things up quickly], or even I would hope beyond them, because of the other experiences I have in life.' R1:453 -61

As noted, students were also acutely aware of the career options available and wider trends and circumstances may thwart or enhance their career trajectories. Hence, another key concern arising from students related to their attractiveness as employment candidates. Older students, in particular, feared that their age may act against them with potential employers as having fewer years of potential service. However, many countered this by arguing that having already had a family and having existing skills in work-life balance management, as well as work experience, could work to the advantage of employers.

## Discussion

Each of the research questions is discussed in turn below.

### How did the students come to be studying dentistry through a graduate entry programme, and what where their motivations for choice of dentistry as a career?

Three main routes to studying dentistry were found - defined by students' motives for, and routes taken to studying dentistry and in terms of time elapsed between their previous and present degree. In short, these can be seen as 'early career changers', 'established career changers' and as pursuing a 'route to specialisation'. In choosing to re-enter study, the graduate entry students interviewed reported mediating between the 'push factors of their previous careers' and the 'pull features of their prospective dental careers'. 'Mediating factors' related to their personal concerns and circumstances. Personal concerns included career fulfilment, quality of life and financial security, factors which emerge from dental students in general [28,34]. 'Pull factors' towards dentistry included financial security, career benefits - such as professional autonomy and flexible working patterns - and career trajectories, again in common with dental students in general [28,34]. 'Push factors' included a lack of career fulfilment, poor working conditions and reimbursement. The opportunity for pursuing specialisation, particularly in Maxillofacial Surgery, was another attractive factor for a dental-related career. Although this Institute has a three-year dental programme for doctors seeking to pursue this surgical training which requires dual qualification in medicine and dentistry [16]. These motivations do not seem to deviate too far from non-graduate entry students' motives [25][28]. There is a shift, however, in the commitments, circumstances and preferences, hence, mediating factors that the students *bring *with them as they embark on a dental career.

With regards to diversification of dental student supply, it can be argued that graduate entry programmes have not yet realised all the potential benefits of such schemes. It has been noted (33) that students who 'park' their previous degree, having originally missed out dental training only to re-apply for graduate entry programmes, have merely delayed entry and represent a similar group. Research from Australia in medical education (34), suggests that graduate entry students rank higher in assessment for clinical skills and bio-scientific knowledge at graduation than 'traditional' entry students. It was found that having a previous degree contributed to stronger acquisition of bio-scientific knowledge, and that age and maturity accounted for better clinical skills. It was interesting then that 'established-career changers' and 'specialisers' in the present study noted the strengths and weaknesses of the skills they had brought to dental practice, and that they felt would become more apparent in practice. Also, the majority of 'early-career changers' stated that their accrued skills had helped them with study-related skills and/or scientific understanding. This would suggest that having a qualification and work experience in another discipline, and the relative addition in age and maturity this infers, informs the students' study and practice of dentistry in a variety of capacities (and frequently positively), regardless of their original motivation for studying dentistry. This suggests that even a small difference in age may diversify skills and interests in dentistry, despite having similar backgrounds to students from the 'traditional' route. Where the background of the student was more disparate (in terms of age and experience) from the traditional route, skills-integration was a key concern evident, as was the motivation to practice dentistry as a public service.

### What factors influence graduate entry students' career expectations for their professional working lives as dentists' in short-term and long-term?

The career expectations of graduate entry students were informed largely by the fit between their motives for study in relation to their perception of the viability and availability of role in the chosen career pathway in dentistry. Many arrived at and mediated their expectations by assessing how different career trajectories matched their original motivation. For example, many respondents who placed being an autonomous practitioner and NHS working at the forefront of their criteria gravitated towards NHS general practice. Others cited the vocational training year as an important opportunity where they believed practical experience could inform their expectations, which fits with the overall remit for foundation years [15,21,43-45]. 'Specialisers', on the other hand, tended to prioritise their long term career goals, and see the NHS as their future working lives and continuing training.

### What factors do students suggest will influence their short-and long-term contribution to the dental profession; and how do their personal concerns, commitments as well as previous career and educational pathway impact on - and shape - their choices?

It was acknowledged by the students that their contribution to NHS dentistry would be mitigated by a range of influences on their possible roles including funding, team-working, workforce skill mix and evidence-based care in relation to their personal motives. The route taken by the students positively informed students' approaches to dentistry, and also posed challenges. Graduate entry students reported bringing practical skills such as study techniques, case management and patient skills - as well as skills in research and working as part of a team (the latter being especially relevant where their degree was healthcare related) to their dental studies and practice. They also felt that the technical knowledge acquired in their previous discipline, and their knowledge of the overall workings and structure of the health service (albeit in a discreet area such as pharmacology, nursing/medicine or bio-medical testing) gave them an adaptable set of skills. Many felt that these skills would become more pertinent in a 'real' practice environment. However, many of those with a biomedical degree felt there was no clear means to integrate their knowledge of their old and new disciplines. Those from other healthcare backgrounds reported being comfortable with patients and working in a team which they considered integral to good practice. Added to this, those planning to specialise in maxillofacial surgery described an ongoing process of weaving both medical and dental disciplines together to fashion their skills and knowledge to the needs of their future discipline. Those intending to specialise sought opportunities that would unite and enhance the sets of skills they brought to their future roles in this highly surgically skilled field. The potential research skills of those who have trained in a bio-medical science are currently an untapped resource in terms of post-qualifying careers.

### What factors within and outside the field on dentistry do graduate entry students believe will impact on their educational and (future) career plans; and how do students appraise, prioritise and arrange these issues and concerns?

It was acknowledged by the students that their contribution to NHS dentistry would be mitigated by funding and reimbursement arrangements, how teams and skills mixes in the workforce and trends in evidence-based medicine influence the roles available for them. In the main students prioritised according to the mediating personal and social factors reported above. Students were also acutely aware of the career options available and how wider trends and circumstances may thwart or enhance their career trajectories; hence, a key concern related to their attractiveness as employment candidates. Older students, in particular, feared that their age may act against them with potential employers as having fewer years of potential service. Many countered this by saying that in many ways having a family and existing skills in work-life balance management held advantages for employers.

### Do any of the issues explored with graduate entry students have utility, or impact, for the future workforce decision-making pertaining to this group or within wider planning?

Although students varied as to whether they wished to work in primary, secondary or tertiary care and in what capacity, all showed a strong commitment to practising in the NHS. In all cases this was rooted in a strong commitment to the principle of dentists having an obligation and responsibility to provide oral healthcare to all. The majority of GEP students also wished to practise outside of London for reasons such as 'giving back' to the community, serving deprived populations and for quality of life reasons; this appears to address one challenge of workforce planning and serve non-metropolitan areas. The present study resonates with earlier work in medical education [43], suggesting that mature graduate entry students bring greater financial and family responsibilities to their studies. This not only has implications for workforce planning, but provides a strong argument for the need to develop organisational structures in training institutions, and wider student support systems, to meet the needs of this group. Similar to the present study, recent research in South Korea [15], found that graduate entry students' motives for studying medicine were more 'altruistic' than those chosen by traditional secondary school results-based selection. The present study suggests that the orientation to public service and serving deprived populations is pronounced in graduate entry students. In publicly funded dental systems, such as NHS provision the UK, policy emphasis (and financial incentives), are being placed on enhancing the patient experience. If the established confidence in patient skills, and drive to improve oral health for all that motivates these students report rings true in practice they may serendipitously acquire a competitive advantage in the workforce. Also, graduate's previous work experience may mean that they are more familiar with the multi-disciplinary nature of modern healthcare practice.

At time of press, this study is the first to contribute to the literature on the views of students completing graduate entry programmes in dentistry. The authors recognise the limitations of this study include the relatively small numbers involved and that they are from one cohort in one school. Another limitation is the setting, as the organisational and policy-related issues are unique to the UK National Health Service and dental training, although many of the aspects resonate with other countries' reports of introducing graduate entry programmes. None-the-less, the findings provide an important introduction to this field of study and should be explored in other settings to determine if similar benefits of graduate-entry programmes can be identified over time and in different settings and the challenges identified are managed. Finally, the longer term implications for the dental workforce in general and the specialty of maxillofacial surgery need to be explored. Further research, both qualitative and quantitative longitudinal studies are required to explore if characteristics such as better patient skills, a drive for altruism and increased interest in specialisation remain salient within this group as they progress in their professional careers.

## Conclusions

Overall, with regards to their course the GEP students interviewed in their final year acknowledged that their course was 'hard work'. Students felt that the unique needs of mature students were not fully considered within the programme; many of those interviewed also noted the difficulties of having to put aside the habits, culture and procedures of their previous discipline. These were, however, minor points for students when compared to the positive aspects they reported. Students reported integrating quickly into the study, and that the course was a quick route into dentistry. Many acknowledged the positive aspects of diversifying the mix of dental students, and that the skills they brought to the dental team were reflected in the wide range of career options available in dentistry. GEP students' motives and routes to study clearly informed the factors that they considered when formulating their career plans. Amongst the personal considerations were family life and work-life balance and having a fulfilling job; hence, the potential to be an independent practitioner appeared to address these concerns for many. For others, where motives related to career specialisation, the potential work in a hospital environment, and the multiple career pathways available, fitted with students' aspirations. Within the context of concerns relating to the current economic climate some students also argued that NHS dentistry represented job security. If the findings of the present study are carried into reality, then diversifying dental student supply by age, previous career and, therefore intrinsically, by life experiences - combined with a system that offers the ability to access an array of varied career options - would generate a good fit between widening the selection criteria for dental study and an engaged workforce.

## Abbreviations

[KCLDI]: King's College London Dental Institute; [NHS]: National Health Service; [GEP]: Graduate Entry Programme; [VT]: Vocational Training

## Competing interests

Three of the authors (JEG, LC and NHFW) are academic staff at King's College Dental Institute. NHFW is Dental Dean and Head of the Dental Institute.

## Authors' contributions

JEG, together with NHFW and LC conceived the overall research programme. PN and JEG developed the protocol and gained ethics committee approval. PN conducted the fieldwork and primary analysis of the data, working with JEG. JEG, PN, LC and NHFW contributed to the paper. All authors reviewed and agreed the final manuscript.

## Pre-publication history

The pre-publication history for this paper can be accessed here:

http://www.biomedcentral.com/1472-6831/11/25/prepub
